# White-light emission from a single heavy atom-free molecule with room temperature phosphorescence, mechanochromism and thermochromism†
†Electronic supplementary information (ESI) available: details of the synthesis; structural information for the compounds (NMR, elemental analysis and mass spectra); and Fig. S1–S32. CCDC 1481207 and 1481208. For ESI and crystallographic data in CIF or other electronic format see DOI: 10.1039/c6sc03038f



**DOI:** 10.1039/c6sc03038f

**Published:** 2016-11-01

**Authors:** Bingjia Xu, Haozhong Wu, Junru Chen, Zhan Yang, Zhiyong Yang, Yuan-Chun Wu, Yi Zhang, Chongjun Jin, Po-Yen Lu, Zhenguo Chi, Siwei Liu, Jiarui Xu, Matthew Aldred

**Affiliations:** a PCFM Lab , GD HPPC Lab , Guangdong Engineering Technology Research Center for High-performance Organic and Polymer Photoelectric Functional Films , State Key Laboratory of Optoelectronic Material and Technologies , School of Chemistry and Chemical Engineering , Sun Yat-Sen University , Guangzhou 510275 , China . Email: yangzhy29@mail.sysu.edu.cn ; Email: ceszy@mail.sysu.edu.cn ; Email: chizhg@mail.sysu.edu.cn ; Fax: +86 20 84112222 ; Tel: +86 20 84112712; b State Key Laboratory of Optoelectronic Material and Technologies , School of Physics and Engineering , Sun Yat-sen University , Guangzhou 510275 , China; c Shenzhen China Star Optoelectronics Technology Co., Ltd , Shenzhen 518107 , China; d Department of Chemistry , Durham University , DH1 3LE , UK

## Abstract

Two heavy atom-free luminophores (SHB_2_t and SDB_2_t) with simple molecular structures have been synthesized *via* Suzuki coupling reactions in which both display white-light emission with prompt fluorescence and room temperature phosphorescence (RTP) in the solid state. The impressive RTP of the luminophores is produced by a synergistic effect of the strong intermolecular hydrogen bonding in addition to the spin–orbit coupling of the sulfonyl oxygen atoms and the moderate singlet-triplet energy gaps (Δ*E*_ST_). These factors facilitate the intersystem crossing (ISC) process to generate triplet excitons in which the molecular conformations become immobilized to effectively suppress radiationless decay. Under the stimuli of mechanical force and solvent vapor, the RTP of SHB_2_t and SDB_2_t can be simply turned off and on by breaking and reforming the robust hydrogen bonding, which leads to remarkable and reversible mechanochromism between white and deep-blue emission. Moreover, two different thermochromic processes have been observed for the pristine and ground samples of SDB_2_t, in which a tricolor switching system between white, deep-blue and blue emission has been successfully achieved through the sequential control of grinding, heating and fuming. From detailed studies we have determined that the mechanism for the thermochromism of SDB_2_t is correlated with the rearrangement of the white-light emitting molecules to a new packing mode without RTP emission.

## Abstract 2

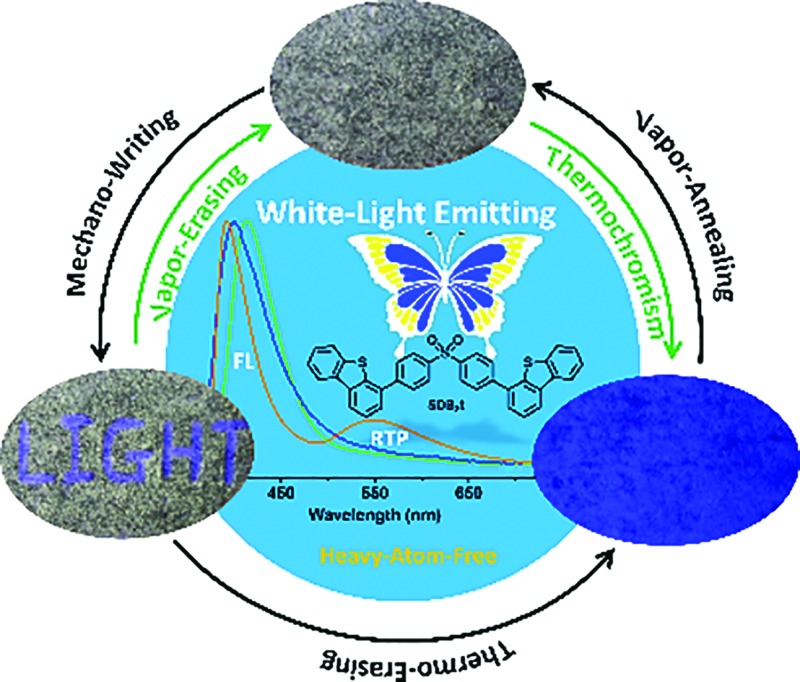
Two heavy atom-free white-light emitting luminophores exhibit fluorescence–phosphorescence dual-emission and are multi-stimuli responsive at room temperature.

## Introduction

White-light emitting materials that have the ability to harness triplet excitons to produce RTP without the assistance of heavy metals and heavy halogen atoms are of great importance from both academic and practical viewpoints because they play a significant role in next generation displays, sensing and solid-state lighting systems.^1^ With regards to white-light emission the majority of the documented materials rely on employing multiple cooperating emitters to create at least dual complementary colors (*e.g.* blue and yellow) to cover the entire visible spectral region.^2^ In comparison with multicomponent sources, white-light emitted directly from a single molecule is acknowledged to be much more attractive since it can largely avoid the drawback of color aging caused by phase-separation and the degradation processes of the combined emitters.^3^ However, due to the intrinsic limitation of Kasha's rule and undesirable energy transfer, white-light emitting single component systems are relatively difficult to be attained and only a few successful examples, such as benzo[*a*]xanthene, pyridone and phenothiazine derivatives, have been reported so far.^3b,4^ Furthermore, most of these luminogens have complicated molecular scaffolds and require sophisticated strategies to synthesise, which greatly impedes their practical uses.^5^ In this context, the design and synthesis of new materials with simple molecular structures that exhibit direct white-light emission in the solid state is of great importance.

Notably, white-light emitting compounds with RTP are superior alternatives to conventional white-light emitting fluorescent luminogens owing to their triplet harvesting features.^3*c*^ They also show efficient responses to external stimuli (*e.g.* pressure, heat, pH and/or solvent vapor) due to their large separation of emission peaks and the sensitivity of the triplet states to conformational rigidity, which offers switchable emission with high contrast color changes in the solid state.^6^ Nevertheless, without heavy metals and/or heavy halogens, spin–orbit coupling of the molecules is commonly in low efficiency, leading to phosphorescence that cannot compete with non-radiative relaxation processes.^1e,7^ As a result, current heavy atom-free organic phosphorescent materials, especially those that exhibit white-light emission and stimuli-responsiveness, are extremely scarce. In fact, organic functional groups with lone electron pairs, for example, aromatic carbonyls and sulfones, are capable of replacing the heavy atoms and work as functional moieties to facilitate electronic coupling and promote ISC from the lowest excited singlet state (S_1_) to the triplet manifold (T_n_).^6b,8^ Meanwhile, by a rational molecular design, such versatile units may also afford strong intermolecular contacts to rigidify the molecular conformations in the crystal structure and then sufficiently suppress the radiationless decay to initiate RTP.^8*a*^ Following this principle, two typical heavy atom-free compounds, namely 9-(4-(phenylsulfonyl)phenyl)-9*H*-carbazole (Cz-DPS, Fig. S1a†) and (4-(9*H*-carbazol-9-yl)phenyl) (phenyl)methanone (Cz-BP, Fig. S1b†), with fluorescence–phosphorescence dual-emission have been developed by our group and Tang *et al.* ^6c,9^ However, the CIE_*x*,*y*_ chromaticity coordinates of these two luminophores are both located in the blue-light region with values of (0.17, 0.09) and (0.17, 0.14) for Cz-DPS and Cz-BP, respectively. Even though within the last three years there has been substantial progress regarding the realization of organic persistent RTP with various emission colors, more attention should be paid to construct heavy atom-free RTP-active compounds with white-light emission and stimuli-responsiveness in terms of their fundamental importance and promising applications.^1e,1f,8c,10^ Therefore, the development of novel white-light emitting materials that are also phosphorescent and stimuli-responsive without heavy metals and heavy halogen atoms remains a top priority in both synthetic chemistry and photophysics.

In this article, we present detailed photophysical investigations of a new family of heavy atom-free butterfly type sulfone derivatives that show white-light emission with RTP. Herein, diphenylsulfone and dibenzothiophene units are employed as building blocks to promote the intersystem crossing (ISC) process and shut down non-radiative channels by the formation of robust hydrogen bonds to immobilize the molecular conformations; both of these effects are beneficial for yielding RTP.^8,11^ Moreover, under the stimuli of force/heat and solvent vapor, the RTP of the as-prepared compounds is facilely turned off and on, thus resulting in remarkable and reversible mechanochromism and thermochromism between white and deep-blue/blue light emission. Such smart white-light emitting luminophores with RTP are impressive and are also potential candidates for future sensors, memory devices and security inks.^12^

## Results and discussion

**Fig. 1:**
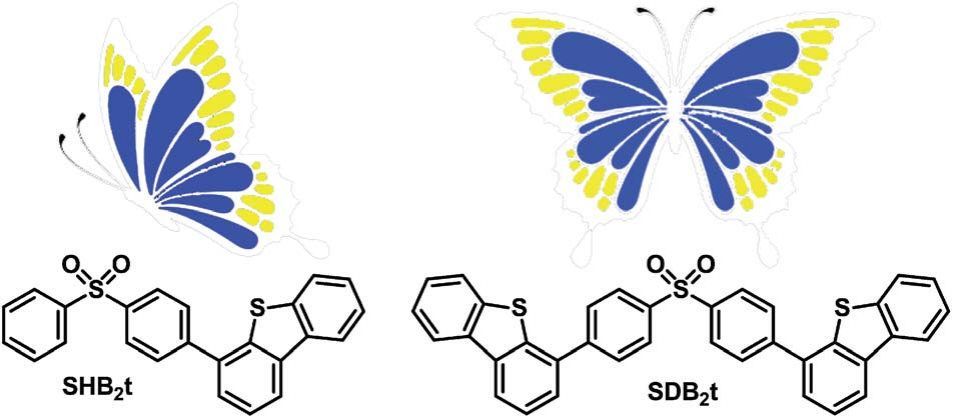
Molecular structures of the heavy atom-free butterfly-type compounds: SHB_2_t and SDB_2_t.

**Fig. 2:**
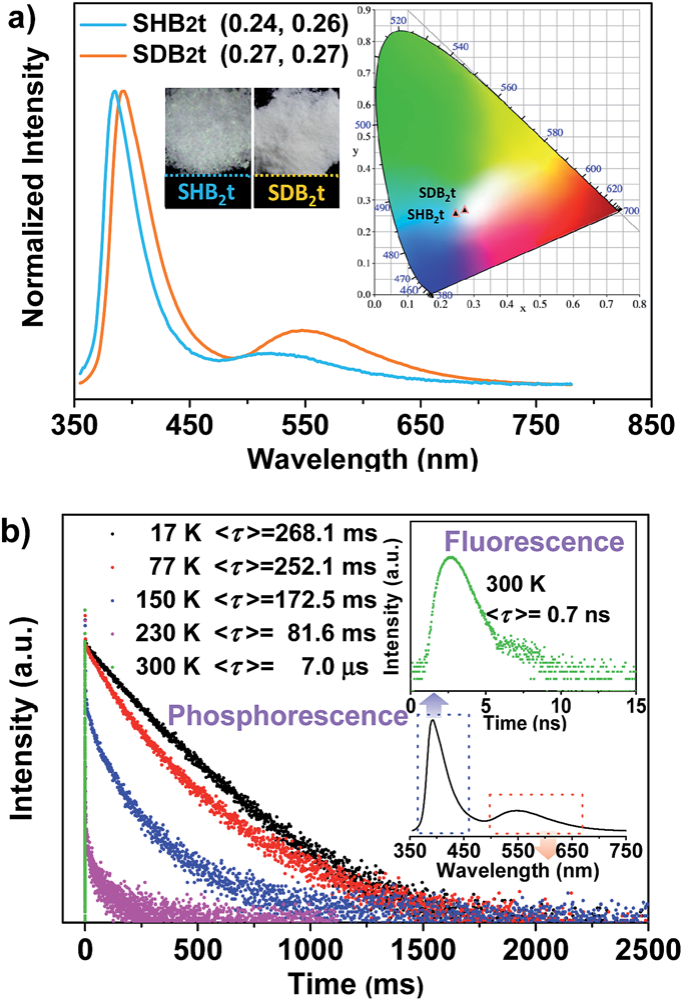
(a) Emission spectra and CIE_*x*,*y*_ coordinates of SHB_2_t and SDB_2_t in the pristine solid state, the insets are the luminescence images of the compounds in the pristine solid state under the irradiation of 365 nm UV light. (b) Emission decay curves of SDB_2_t in the solid state at different temperatures.

The chemical synthesis of the butterfly-type luminogens was accomplished by simply incorporating one or two dibenzothiophene units at the *para* position(s) of the diphenylsulfone core *via* Suzuki coupling reactions (Fig. 1, Scheme S1 and Fig. S25–S32†). As depicted in Fig. S2,† the target compounds, namely SHB_2_t and SDB_2_t, exhibit similar UV-vis absorption and photoluminescence (PL) emission spectra profiles in air-saturated dilute solutions owing to their identical effective chromophoric components. The absorption peaks at 292 nm and 335 nm are assigned to ^1^π–π* and intramolecular charge transfer (CT) transitions, respectively. On the other hand, the single broad emission peaks at 386 and 388 nm for SHB_2_t (*Φ*_sl_ = 9%) and SDB_2_t (*Φ*_sl_ = 10%), respectively, might originate from the radiative decay of the CT state due to their dipolar nature. Further evidence for this viewpoint is provided by the positive solvatochromism of SHB_2_t and SDB_2_t in solutions with different polarities (Fig. S3 and S4†). The PL spectra of these two compounds in oxygen-free solutions were also investigated (Fig. S5†) and the results agreed well with those obtained from air-saturated ones, indicating that the triplet energy loss of these two compounds in solution is mainly caused by the intermolecular interactions between the compound and the surrounding solvent molecules. In the solid state, both SHB_2_t (*Φ*_s_ = 7%) and SDB_2_t (*Φ*_s_ = 13%) are dual-emissive with an extra broad emission band that peaks at 519 and 549 nm (Fig. 2a), respectively. The CIE_*x*,*y*_ coordinates of SHB_2_t and SDB_2_t are calculated to be (0.24, 0.26) and (0.27, 0.27), which confirms their white-light emitting properties. In comparison with SHB_2_t, the two emission bands of SDB_2_t are found to be bathochromically shifted, which could be tentatively ascribed to the enhanced polarization effect caused by the stronger intermolecular dipole interactions among the molecules of SDB_2_t with larger dipole moments. Meanwhile, it is worth noting that the white-light emitting compounds of SHB_2_t and SDB_2_t in the solid state are very stable and can be stored in air for more than one year.

Transient PL decay was subsequently carried out at room temperature (300 K) to investigate the excited-state nature of SHB_2_t and SDB_2_t (Fig. 2b and S6†). The emission bands in the blue-violet region exhibit prompt decays with short lifetimes of 0.4 ns (SHB_2_t) and 0.7 ns (SDB_2_t) and are certainly attributed to fluorescence. However, the yellow emission bands display slow decays with long lifetimes of 35.6 ms (SHB_2_t) and 7.0 μs (SDB_2_t) and are ascribed to phosphorescence. The phosphorescence characteristics of the long-lived yellow emission bands of SHB_2_t and SDB_2_t are further validated by the temperature dependent emission decay, which shows a continuous decrease in lifetime from 244.8 ms (SHB_2_t) and 268.1 ms (SDB_2_t) at 17 K to the values noted above at 300 K. Therefore, these results unambiguously corroborate that both SHB_2_t and SDB_2_t are white-light emitting luminophores with RTP. The RTP profiles of these two compounds in the solid state are in accordance with the emission spectra in dimethyltetrahydrofuran solution at 77 K, which are also broad and structureless (Fig. S7†), indicating that they probably originate from the ^3^CT state of a single molecule. Accordingly, the Δ*E*_ST_ of SHB_2_t and SDB_2_t can be estimated from the onset of their two CT emission bands and are calculated to be 0.68 and 0.65 eV, respectively.^13^ The moderate Δ*E*_ST_ values combined with the strong spin–orbit coupling at the sulfonyl oxygen atoms allow intrinsic triplet generation through ISC and activate the RTP.^14^ The sharp peaks in the powder X-ray diffraction (XRD) patterns that are depicted in Fig. S8† illustrate that the as-prepared white-light emitting samples of SHB_2_t and SDB_2_t are mainly composed of microcrystals. In other words, the emergence of the yellow phosphorescence band might be induced by crystallization of the compound.

**Fig. 3:**
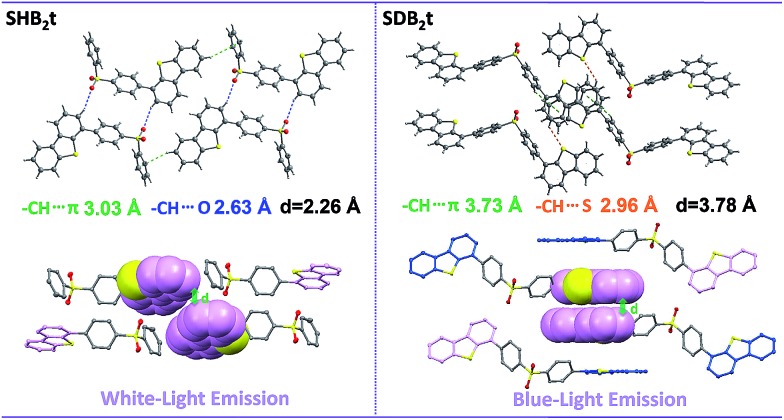
Intermolecular interactions and molecular stacking of SHB_2_t and SDB_2_t in single crystal structures.

Considering that both SHB_2_t and SDB_2_t are composed of a diphenylsulfone unit and dibenzothiophene moieties, the photophysical properties of these two compounds might be related to the dihedral angle(s) between the dibenzothiophene unit and its adjacent phenyl ring. To understand the effects of these two fragments and gain more insight into their RTP, white-light emitting single crystals of SHB_2_t and, unexpectedly, blue-light emitting single crystals of SDB_2_t without the yellow phosphorescence band were obtained. The PL emission spectra of these two single crystals are presented in Fig. S9.† Although the white-light single crystal of SDB_2_t is difficult to achieve owing to its high tendency to self-assemble into fibers, the analysis of the blue-light emitting single crystal should also provide useful information to decipher the origin of their RTP. The popular B3LYP density functional theory was then used to calculate the electronic transition characteristics of these two compounds at the 6-31G(d,p) level based on their ground state geometries in single crystals. SHB_2_t and SDB_2_t show large dipole moments in the ground state and the values are determined to be 6.21 and 6.78 debye, respectively. As depicted in Table S1,† the simulated Δ*E*_ST_ value of SHB_2_t (0.73 eV) is also moderate and is larger than that of SDB_2_t (0.60 eV), which is in accordance with the experimental results. Furthermore, the intramolecular CT characteristics of SHB_2_t and SDB_2_t are substantiated (Fig. S10†). The ^1^CT absorption of SHB_2_t is evaluated to be 321 nm (3.86 eV, from HOMO to LUMO, *f* = 0.0983), which is close to the experimental assignment (340 nm, Fig. S11†). The ^1^ππ* absorption of SHB_2_t mainly originates from the HOMO–1 to LUMO+1 transition and is calculated to be 286 nm (4.34 eV, *f* = 0.1634). This result also corresponds qualitatively to the experimental one (295 nm). The ^1^ππ* and ^1^CT absorptions of SDB_2_t in the blue-light emitting single crystal are simulated to be 319 nm (3.88 eV, from HOMO to LUMO+1, *f* = 0.0468) and 346 nm (3.58 eV, from HOMO to LUMO, *f* = 0.3093), respectively. As depicted in Fig. S11,† the pristine white-light emitting microcrystals of SDB_2_t and SHB_2_t show similar absorption profiles, indicating that the molecular conjugation in the ground state and the dihedral angles between the dibenzothiophene groups and their neighboring phenyl rings for the SDB_2_t molecule in a microcrystal are probably close to those of SHB_2_t. The experimental XRD pattern of the white-light emitting microcrystal of SHB_2_t agrees well with the simulated XRD pattern of the white-light emitting single crystal (Fig. S12a†), suggesting that the microcrystal of SHB_2_t adopts the same molecular arrangement as that of the single crystal. However, in the simulated XRD pattern of the blue-light emitting single crystal of SDB_2_t, some of the intense and sharp diffraction peaks show a slight shift in position in comparison to the experimental pattern of the white-light emitting microcrystal (Fig. S12b†). That is, the molecular packing in the microcrystal is probably different from the single crystal for SDB_2_t. In the unit cells of the SHB_2_t single crystal, the dibenzothiophene group exhibits a dihedral angle of 56.47° to its neighboring phenyl ring, leading to strong C–H···O hydrogen bonds with lengths of 2.63 Å and tight molecular packing with an intermolecular distance of 2.26 Å in the crystal structure (Fig. 3 and S13†). In contrast, the dihedral angles in the blue-light emitting SDB_2_t single crystal are determined to be 47.71°, which is much smaller than those observed for the white-light emitting SHB_2_t. As a result, strong C–H···O hydrogen bonds and a compact molecular arrangement are superseded by the weak C–H···S contacts (2.96 Å) and a loose molecular stacking mode with a larger intermolecular distance (3.78 Å). In other words, compared to in the blue-light emitting single crystal, the molecular conformation of SDB_2_t in the white-light emitting microcrystal may be more twisted, thus resulting in stronger intermolecular contacts and more compact molecular packing. The intense intermolecular interactions, namely C–H···O hydrogen bonds, efficiently impede the vibrations and rotations of the molecular fragments to suppress the radiationless decay of the triplet excitons and finally yielding the RTP.

It is noteworthy that the yellow phosphorescence bands of the microcrystals of SHB_2_t and SDB_2_t are gradually reduced by applying mechanical force to their pristine samples (Fig. S14† and 4a). Accompanying these changes, the PL maxima in the blue-violet region shifts from 385 nm (SHB_2_t) and 392 nm (SDB_2_t) to 400 nm (SHB_2_t, *Φ*_s_ = 7%) and 414 nm (SDB_2_t, *Φ*_s_ = 9%). Consequently, the CIE_*x*,*y*_ coordinates of all the points converted from the proceeding emission alteration lie on two straight lines with satisfying correlation coefficients of 0.9942 and 0.9979. This suggests that the emission colors of SHB_2_t and SDB_2_t can be linearly tunable from white to deep-blue with CIE_*x*,*y*_ values of (0.17, 0.06) and (0.18, 0.08), respectively, even under the mild treatment of hand grinding. Although the thermograms for the samples of SHB_2_t change very little upon grinding (Fig. S15†), the diffraction peaks in the experimental XRD patterns for the powders of SHB_2_t and SDB_2_t become less defined and more diffuse as the grinding progresses (Fig. S16†). The effect of oxygen in the atmosphere on the luminescence properties is negligible (Fig. S17†). Meanwhile, the solid state UV-vis absorption spectra of the ground samples of both SHB_2_t and SDB_2_t slightly red-shift in comparison to those of the original powders (Fig. S18†). Therefore, in view of the single crystal analysis results noted above, the mechanochromism of SHB_2_t and SDB_2_t is believed to be associated with a ‘crystalline to amorphous transition’ and a localized conformational planarization, which destroys the strong intermolecular interactions and opens up the radiationless decay channels to quench the RTP. By fuming with dichloromethane (DCM) vapor, the yellow phosphorescence bands of SHB_2_t and SDB_2_t arise again (Fig. S19†) and the emission colors are restored to white, exhibiting a reversible mechanochromism. Further evidence is provided by the recovery of the diffractograms and the thermograms (Fig. S20 and S21†), implying that the crystal structures with strong intermolecular contacts are probably reconstructed with the assistance of the DCM vapor.

**Fig. 4:**
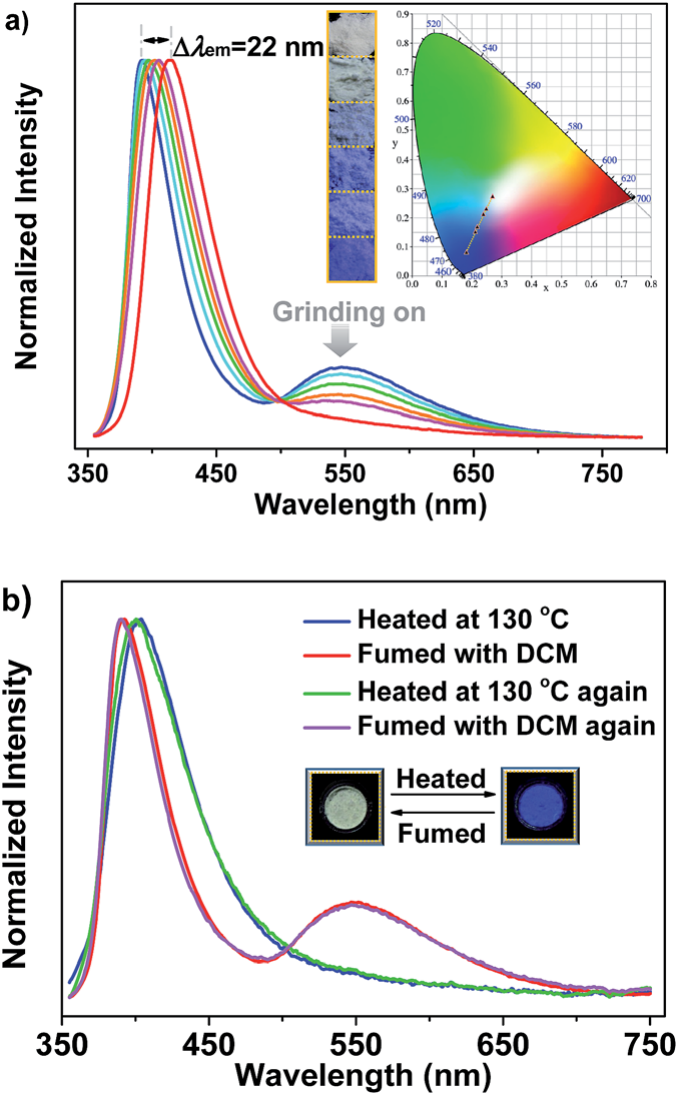
(a) PL emission spectra of microcrystals of SDB_2_t upon grinding, the insets are the luminescence images of the samples and their corresponding CIE_*x*,*y*_ coordinates. (b) PL emission spectra of microcrystals of SDB_2_t upon heating at 130 °C for about 1 min and fuming in DCM vapor for about 20 min, the insets demonstrate a luminescence switching process between the white-light and the blue-light.

Careful investigation reveals that the pristine sample of SDB_2_t displays two endothermic transitions in the differential scanning calorimetry (DSC) curve (Fig. S15b†). The latter sharp one at 250 °C is attributed to the melting point temperature of crystals, whereas the former broad one at 116 °C is probably correlated with the transformation of the crystal structure. To verify this hypothesis, XRD and DSC measurements were carried out to determine the phase characteristics of the annealed sample compared to the original SDB_2_t. As shown in Fig. S22a,† the entirely distinct diffraction peaks in the XRD pattern strongly identifies a new molecular stacking mode of the thermally annealed powder. The molecular packing of the thermally annealed sample is also different from the as-obtained blue-emitting single crystal of SDB_2_t, as shown by their dissimilar XRD patterns (Fig. S12b and S22a†). Moreover, the identical endothermic peaks at 250 °C in the DSC curves further show that the broad transition at 116 °C comes from the rearrangement of the molecules (Fig. S22b†). Intriguingly, we have found that the thermal annealing also triggers a change in the emission color, from white to blue with CIE_*x*,*y*_ coordinates of (0.18, 0.11), showing notable thermochromism for SDB_2_t, which is seldom reported for white-light emitting luminophores (Fig. 4b). In the PL emission spectrum of the thermally annealed sample (*Φ*_s_ = 8%), the yellow phosphorescence band disappeared and the blue-violet fluorescent peak shifted to 402 nm. From the relatively broad diffraction peaks, these variations for the annealed SDB_2_t may result from its loose molecular packing, which activates the vibrational loss of triplets and hinders the emission of RTP. Likewise, the blue emission of the thermally annealed sample is able to be reversed back to white with the aid of DCM vapor (solvent annealing). This experimental result is fully supported by the recoveries in the PL emission spectra as well as the XRD patterns and DSC curves of the fumed samples.

**Fig. 5:**
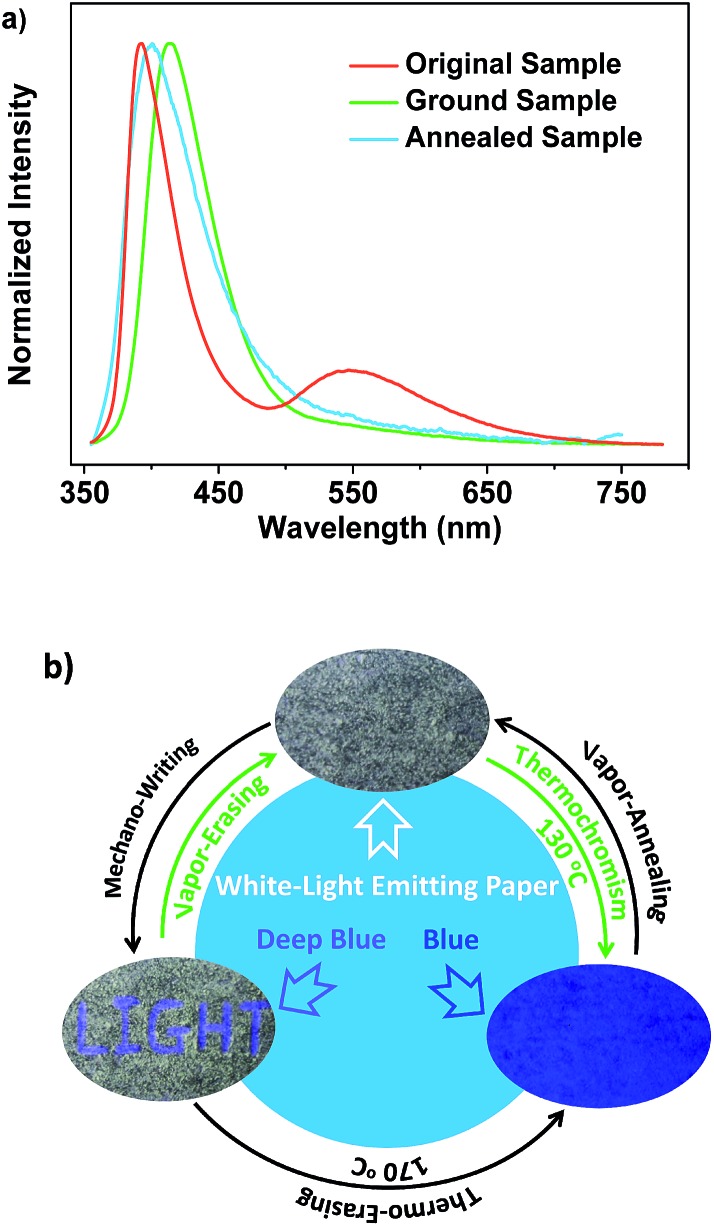
(a) PL emission spectra of SDB_2_t in different states. (b) Emission switching of SDB_2_t in different states (the emission switching was carried out on a piece of white-light emitting ‘smart paper’ fabricated by spreading the white-light emitting powder of SDB_2_t on a piece of filter paper).

Noticeably, upon grinding, the endothermic transition at 116 °C in the DSC curve of SDB_2_t gradually disappeared and an exothermic one at 157 °C emerged (Fig. S15b†). However, the melting peaks observed in the thermograms of the ground samples correspond well with those of the original and the thermally annealed powders. To clarify this issue, the well-ground powder of SDB_2_t with deep-blue emission was heated at 170 °C for about 2 min and a PL emission spectrum was recorded. The PL emission profile of the thermally annealed sample from the well-ground powder overlapped that of the thermally annealed sample from the pristine microcrystals, suggesting that another thermochromic process from deep-blue to blue emission takes place for SDB_2_t (Fig. S23†). This phenomenon is synchronously corroborated by their similar XRD patterns and DSC curves (Fig. S24†). Although the thermochromic process from the well-ground powder to the thermally annealed sample is irreversible, tricolor emission switching between the white, deep-blue and blue colors has been successfully achieved for SDB_2_t through the sequential control of grinding, heating and fuming. It should be noted that any of the ground samples of SDB_2_t with the CIE_*x*,*y*_ coordinates located on the straight line between the white and the deep-blue light is actually convertible to the blue light state by annealing at 170 °C due to the facile transformation of the crystal structure. Such unique luminescence switching behaviors of SDB_2_t triggered by mechanical/thermal stimuli, therefore, enable us to design a white-light smart system based on fluorescence–phosphorescence. As demonstrated in Fig. 5, mechanowriting of the text ‘LIGHT’ and color switching to the blue-light emitting paper are both easily implemented on a piece of recyclable white-light emitting paper under UV-irradiation, which demonstrates SDB_2_t to be a promising anti-counterfeit material.

## Conclusions

In summary, two heavy atom-free white-light emitting luminophores, SHB_2_t and SDB_2_t, which exhibit fluorescence–phosphorescence dual-emission and are multi-stimuli responsive at room temperature have been synthesized for the first time. The generation of RTP for these compounds has been identified to be associated with the synergistic effect of the strong intermolecular hydrogen bonding, as well as the electronic coupling facilitated by the diphenylsulfone and the moderate Δ*E*_ST_ values. This suggests a direction for the molecular design of novel white-light emitting compounds with RTP in the absence of heavy metals and/or heavy halogen atoms. Furthermore, reversible mechanochromism and thermochromism between white and deep-blue and/or blue emission have been achieved in this system by turning on and off the RTP of the compounds using external stimuli. Owing to their simple molecular structures and straightforward syntheses, SHB_2_t and SDB_2_t may have the potential for applications in displays, sensors, memory devices, and lighting devices.
