# Supplementary material for: White-light emission from a single heavy atom-free molecule with room temperature phosphorescence, mechanochromism and thermochromism
Source: Chem Sci. 2016 Nov 1;8(3):1909–14. doi: 10.1039/c6sc03038f (PMC5430137; doi:10.1039/c6sc03038f)
Supplement: Supplementary file 1 [file SC-008-C6SC03038F-s001.pdf]

Electronic Supplementary Material (ESI) for Chemical Science.
This journal is © The Royal Society of Chemistry 2016

Electronic Supporting Information

White-light emission from a single heavy-atom-free molecule with room temperature phosphorescence, mechanochromism and thermochromism

Bingjia Xu, Haozhong Wu, Junru Chen, Zhan Yang, Zhiyong Yang,* Yuan-Chun Wu, Yi Zhang,* Chongjun Jin, Po-Yen Lu, Zhenguo Chi,* Siwei Liu, Jiarui Xu, and Matthew Aldred

1. General experimental procedures

Materials. Bis(4-bromophenyl) sulfone, benzenesulfonyl chloride, bromobenzene, iron chloride, dibenzo[*b*,*d*]thiophen-4-ylboronic acid and tetrakis(triphenylphosphine) palladium(0) purchased from Alfa Aesar were used as received. 1-bromo-4-(phenylsulfonyl)benzene (SBr)[1] was synthesized according to the literature method. All other reagents and solvents were purchased with analytical grade from Guangzhou Jincheng Company (China) and used without further purification.

Characterizations. Proton and carbon NMR (^{1}H NMR and ^{13}C NMR) spectra were measured on a Bruker AVANCE 400 spectrometer and a Bruker AVANCE III spectrometer ($CDCl_3$, tetramethylsilane as the internal standard). The elemental analysis was performed with a Vario EL analyzer. The mass spectra were measured using thermospectrometers (DSQ & MAT95XP-HRMS). The UV-visible absorption spectra were determined on a Hitachi U-3900 spectrophotometer. The PL spectra were measured with a Shimadzu RF-5301 PC spectrometer or on an Ocean Optics Maya Pro2000 with a 310 nm Rhinospectrum RhinoLED as the excitation source. Wide-angle XRD measurements were performed at 293 K using a X-ray diffractometer (Smartlab, Rigaku Co.) with an X-ray source of Cu Kα (λ= 0.15406 nm) at 40 kV and 30 mA at a scan rate of 4° (2ϑ)/min. The time-resolved emission decay behaviors were obtained using a spectrometer (FluoroLog-TCSPC) from HORIBA Instruments. The thermal behaviors were determined by differential scanning calorimetry (DSC) at heating rate of 10 ^{o}C/min under N_2 atmosphere using a NETZSCH thermal analyzer (DSC 204F1).The quantum chemistry calculation was performed at the B3LYP/6-31+G (d, p) level of theory using the DFT method in the Gaussian 09 software.

While the white-light emitting single crystals of SHB_2t that were suitable for single crystal X-ray structural analysis were directly picked out from the as-prepared crystalline powder, the blue-light emitting single crystals of SDB_2t were isolated from a mixture of *n*-hexane and CH_2Cl_2 through the method of solvent evaporation. The single-crystal X-ray diffraction data for the compounds were collected from an Agilent Technologies Gemini A Ultra system with Cu-Kα radiation (λ = 1.54178 Å) at 293(10) K. The structures were solved using direct methods following the difference Fourier syntheses. All non-hydrogen atoms were anisotropically refined through least-squares on F^2 using the SHELXTL program suite. The anisotropic thermal parameters were assigned to all non-hydrogen atoms. The hydrogen atoms attached to carbon were placed in idealized positions and refined using a riding model to the atom from which they were attached. The pictures of the three structures were produced using Diamond 3.2. CCDC 1481207 and 1481208 contain the supplementary crystallographic data for

this paper.

2. Synthesis

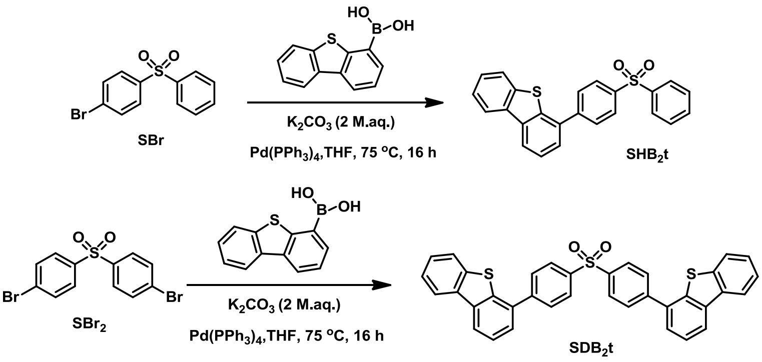

Scheme S1. Synthetic routes of the target compounds.

General procedures for the synthesis of the target compounds.

Dibenzo[*b,d*]thiophen-4-ylboronic acid (7.50 mmol) and 1-bromo-4-(phenylsulfonyl)benzene (SBr) (1.49 g, 5.00 mmol) or 4,4'-sulfonylbis(bromobenzene) (SBr_2) (0.94 g, 2.50 mmol) were dissolved in tetrahydrofuran (THF) (40 mL), and then 2 M. aqueous K_2CO_3 solution (5.6 ml) was added. The mixture was stirred for 30 min under an argon atmosphere at room temperature. Then $Pd(PPh_3)_4$ catalyst was added, and the reaction mixture was stirred at 75 ˚C for 16 h. After cooling to room temperature, the product was concentrated and purified by silica gel column chromatography with dichloromethane/*n*-hexane (v/v=4:5) as eluent.

4-(4-(phenylsulfonyl)phenyl)dibenzo[b,d]thiophene (SHB$_2$t). Compound SHB_2t was recrystallized from the mixture of dichloromethane/*n*-hexane and obtained as a white crystalline solid in 76% yield (1.52 g). ^1H NMR (500 MHz, $CDCl_3$, TMS) δ (ppm): 8.25-8.19 (d, *J*=7.4 Hz, 2 H); 8.14-8.09 (d, *J*=8.3 Hz, 2 H); 8.09-8.02 (d, *J*=7.7 Hz, 2 H); 7.94-7.89 (d, *J*=8.2 Hz, 2 H); 7.88-7.83 (m, 1 H); 7.67-7.56 (ddd, *J*=12.4, 11.0, 6.0 Hz, 4 H); 7.55-7.46 (dt, *J*=13.4, 6.5 Hz, 3 H). ^{13}C NMR (100 MHz, $CDCl_3$, TMS) δ (ppm): 146.94, 142.97, 142.34, 139.70, 136.33, 134.67, 130.52, 129.18, 128.43, 126.03, 124.01, 123.19, 122.88. EI-MS, m/z: $[M]^+$ 400; calcd for $C_{24}H_{16}O_2S_2$ 400. HRMS, m/z: $[M]^+$ 400.0588; calcd for $C_{24}H_{16}O_2S_2$ 400.0592. Anal. Calc. for $C_{24}H_{16}O_2S_2$: C 71.97%, H 4.03%, S 16.01%; found: C 71.91%, H 4.08%, S 16.05%.

4,4'-(sulfonylbis(4,1-phenylene))didibenzo[b,d]thiophene (SDB$_2$t). Compound SDB_2t was recrystallized from the mixture of dichloromethane/ethanol and obtained as a white powder in 68% yield (0.99 g). ^1H NMR (500 MHz, $CDCl_3$, TMS) δ (ppm): 8.24-8.19 (ddd, *J*=6.3, 5.3, 1.2 Hz, 4 H); 8.19-8.15 (m, 4 H); 7.99-7.91 (m, 4 H); 7.87-7.81 (m, 2 H); 7.62-7.56 (dd, *J*=9.6, 5.7 Hz, 2 H); 7.53-7.45 (m, 6 H). ^{13}C NMR (125 MHz, $CDCl_3$, TMS) δ (ppm): 149.82, 142.22, 140.81, 138.44, 133.62, 132.35, 129.42, 128.74, 127.41, 126.37, 126.32, 115.88, 100.39. EI-MS, m/z: $[M]^+$ 582; calcd for $C_{36}H_{22}O_2S_3$ 582. HRMS, m/z: $[M]^+$ 582.0774; calcd for $C_{36}H_{22}O_2S_3$ 582.0782. Anal. Calc. for $C_{36}H_{22}O_2S_3$: C 74.20%, H 3.81%, S 16.51%; found: C 74.23%, H 3.83%, S 16.55%.

Reference

1 M. Alexander, A. Khandekar and S. Samant, *J. Mol. Cata. A-Chem.*, 2004, **223**, 75.

3. Figures

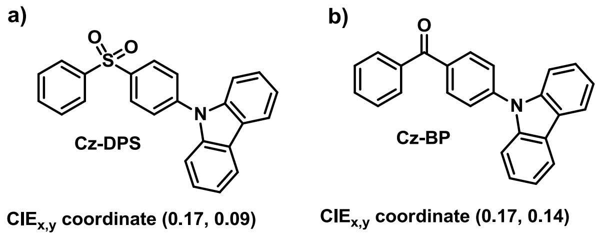

Fig. S1 Molecular structures of the two typical fluorescence-phosphorescence dual-emissive compounds.

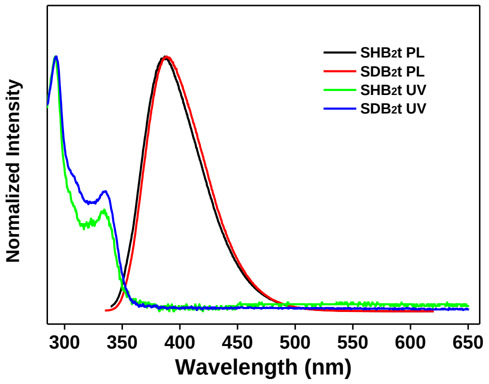

Fig. S2 UV-vis absorption and PL spectra of SHB_2t and SDB_2t in tetrahydrofuran (THF) solutions.

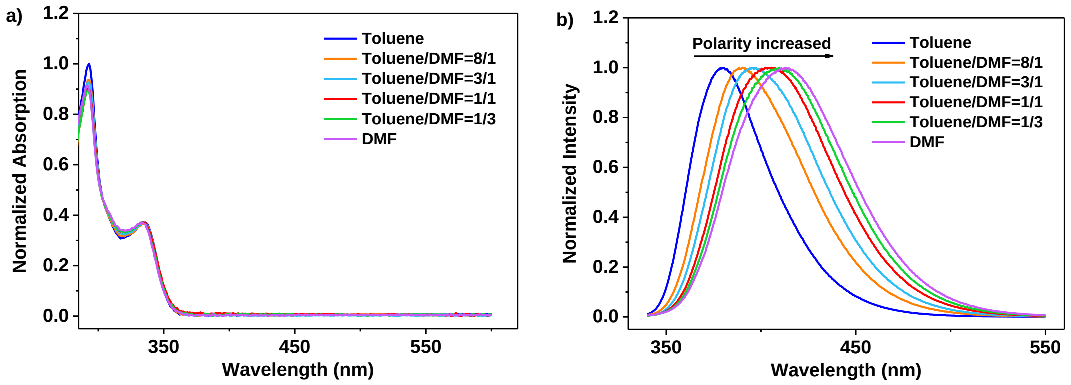

Fig. S3 Changes of a) UV-vis absorption and b) PL spectra for SHB_2t in the mixtures of toluene and DMF with different volume ratio.

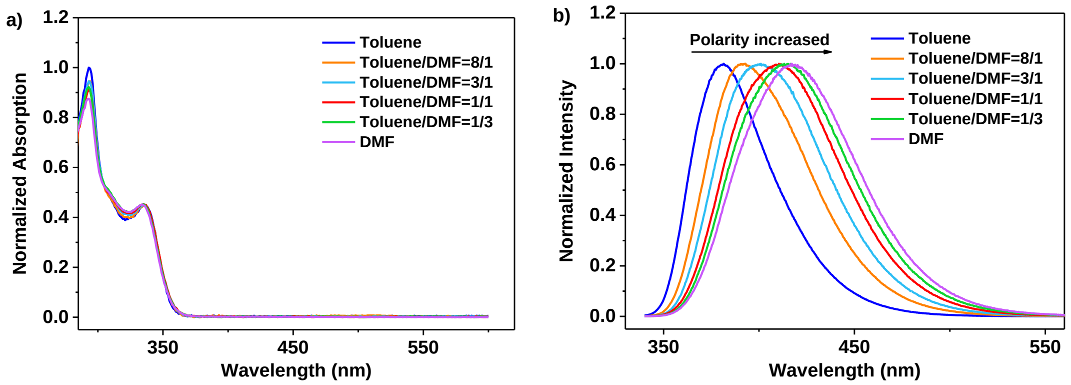

Fig. S4 Changes of a) UV-vis absorption and b) PL spectra for SDB_2t in the mixtures of toluene and DMF with different volume ratio.

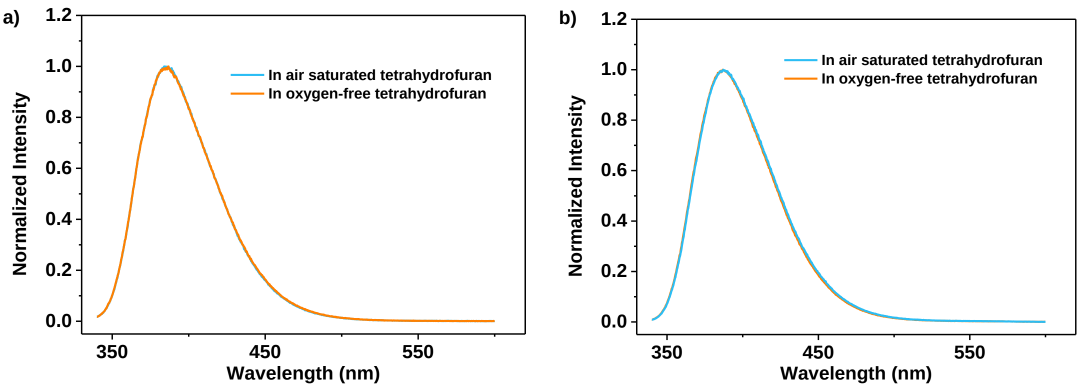

Fig. S5 PL spectra of a) SHB_2t and b) SDB_2t in THF at different conditions (10 μM).

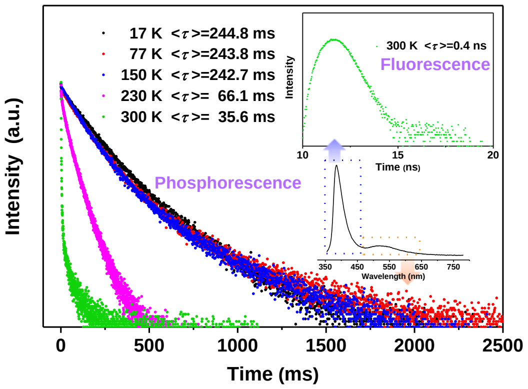

Fig. S6 Emission decay curves of SHB_2t in the solid state at different temperatures.

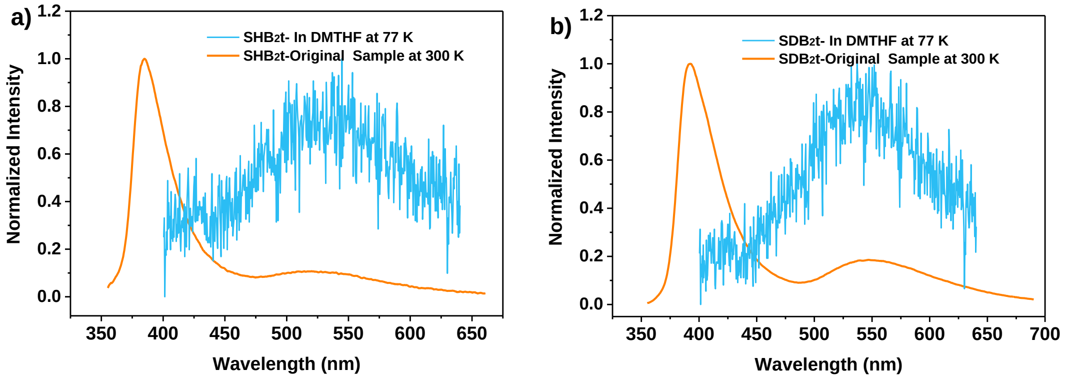

Fig. S7 PL emission spectra of a) SHB_2t and b) SDB_2t in dimethyltetrahydrofuran (DMTHF, 10 μM) and in the solid state.

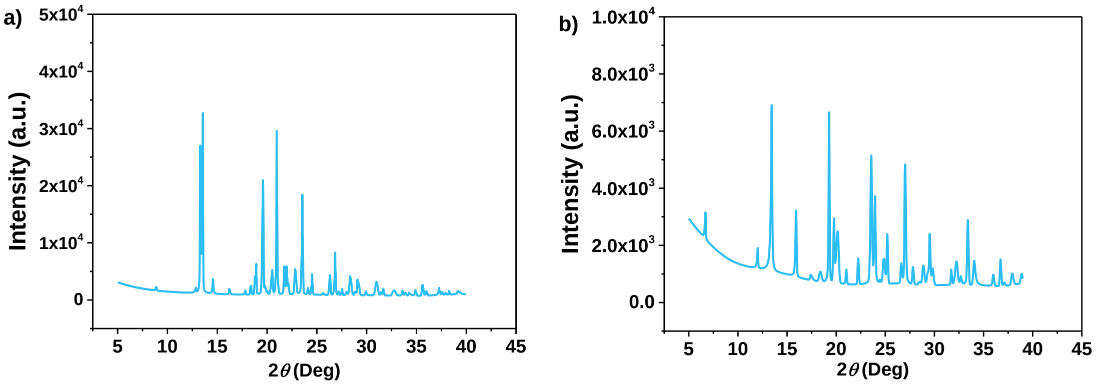

Fig. S8 XRD patterns of the pristine samples of a) SHB_2t and b) SDB_2t.

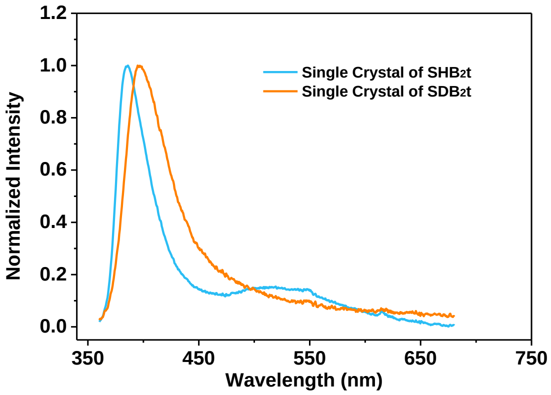

Fig. S9 PL spectra of the single crystals of SHB_2t and SDB_2t.

Table S1 Calculated energy levels of the excited states of SHB_2t and SDB_2t from their single crystal structures

Compound	Excited State	Energy Level (eV)	Wavelength (nm)	oscillator strength (f)
**SHB_2t**	T_1	3.13	396	0.0000
	T_2	3.40	365	0.0000
	S_1	3.86	321	0.0983
	S_2	4.17	297	0.0076
**SDB_2t**	T_1	2.98	416	0.0000
	T_2	3.03	409	0.0000
	S_1	3.58	346	0.3093
	S_2	3.64	341	0.0171

Note: DFT based on B3LYP is one of the most popular methods for the simulation of electronic transition characteristics of organic compounds, including the molecules with prompt fluorescence, room temperature phosphorescence (RTP) or thermally activated delayed fluorescence (TADF).[1] In some cases, the calculated data from B3LYP could also make a good prediction and/or fit the experimental results well.[2] Of course, for characterizing compounds with CT states, range-separated hybrid functions sometimes would be better choices in comparison to B3LYP. However, in this system, it seems not the case. Herein, CAM-B3LYP, one of the typical range-separated hybrid functions, has been used to recalculate the electronic transition features of the compounds. The ΔE_{ST} values of SHB_2t and SDB_2t are respectively determined to be 1.43 eV and 1.37 eV, which are extremely large and are both far away from the experimental ones. The ^{1}CT absorptions of SHB_2t and SDB_2t in single crystals are calculated to be 4.43 eV (280 nm) and 4.28 eV (290 nm). Obviously, these results do not match the experimental assignments (340 nm, Fig. S11) as well. Thus, it seems that CAM-B3LYP is not a suitable function for this system.

References

1 *(a)* R. Yoshii, A. Hirose, K. Tanaka, and Y. Chujo, *J. Am. Chem. Soc.*, 2014, **136**, 18131; *(b)* Z. Yang, Z. Mao, X. Zhang, D. Ou, Y. Mu, Y. Zhang, C. Zhao, S. Liu, Z. Chi, J. Xu, Y.-C. Wu, P.-Y. Lu, A. Lien, and M. R. Bryce, *Angew. Chem. Int. Ed.*, 2016, **55**, 2181; *(c)* Y.-J. Shiu, Y.-C. Cheng, W.-L. Tsai, C.-C. Wu, C.-T. Chao, C.-W. Lu, Y. Chi, Y.-T. Chen, S.-H. Liu, and P.-T. Chou, *Angew. Chem.*, 2016, **128**, 3069; *(d)* M. Shimada, M. Tsuchiya, R. Sakamoto, Y. Yamanoi, E. Nishibori, K. Sugimoto, and H. Nishihara, *Angew. Chem.*, 2016, **128**, 3074; (*e*) B. Xu, Y. Mu, Z. Mao, Z. Xie, H. Wu, Y. Zhang, C. Jin, Z. Chi, S. Liu, J. Xu, Y.-C. Wu, P.-Y. Lu, A. Lien and M. R. Bryce, *Chem. Sci.*, 2016, **7**, 2201.

2 *(a)* Q. Zhang, J. Li, K. Shizu, S. Huang, S. Hirata, H. Miyazaki, and C. Adachi, *J. Am. Chem. Soc.*, 2012, **134**, 14706; *(b)* H. Wang, L. Xie, Q. Peng, L. Meng, Y. Wang, Y. Yi, and P. Wang, *Adv. Mater.*, 2014, **26**, 5198; *(c)* S. Y. Lee, T. Yasuda, Y. S. Yang, Q. Zhang, and C. Adachi, *Angew. Chem. Int. Ed.*, 2014, **53**, 6402; *(d)* G. Méhes, H. Nomura, Q. Zhang, T. Nakagawa, and C. Adachi, *Angew. Chem. Int. Ed.*, 2012, **51**, 11311.

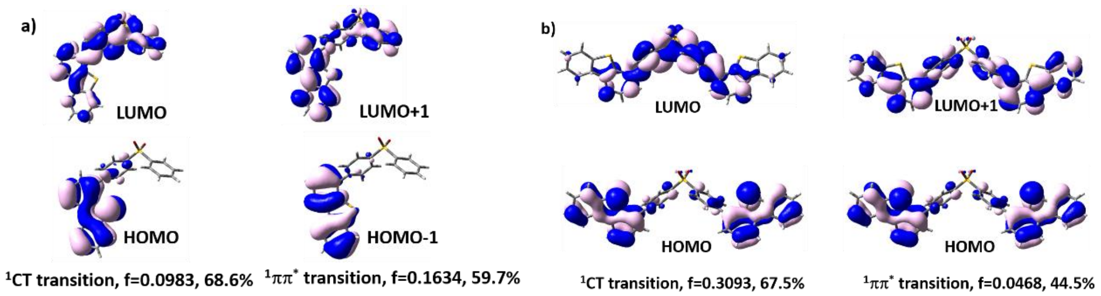

Fig. S10 Calculated electronic transition orbits and the corresponding oscillator strengths (f) of a) SHB_2t and b) SDB_2t from their single crystal structures.

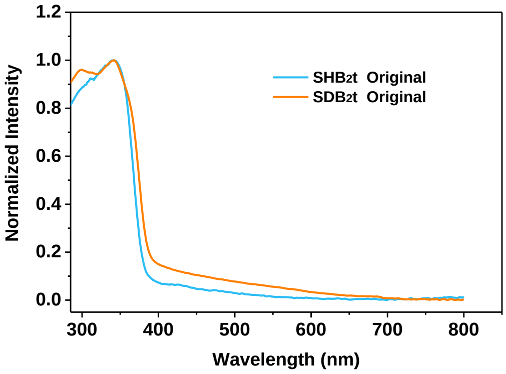

Fig. S11 UV-vis absorption spectra of the original white-light emitting microcrystals for SHB_2t and SDB_2t.

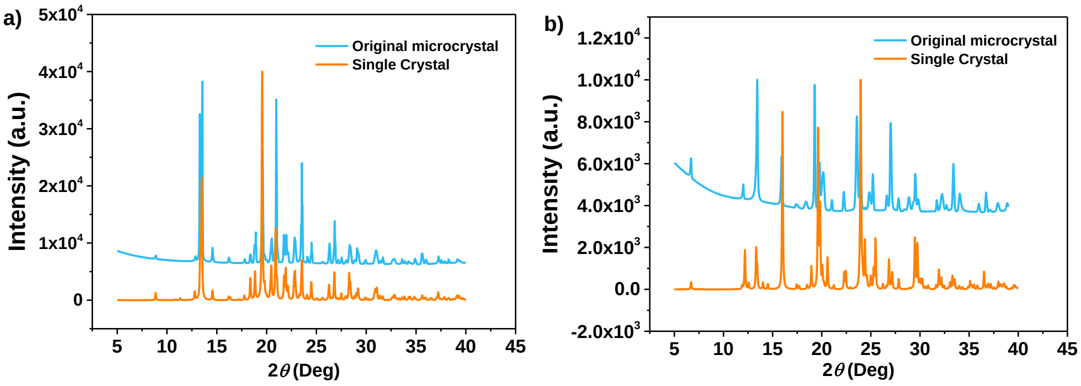

Fig. S12 Experimental XRD patterns of the original microcrystals and simulated XRD patterns of the single crystals of a) SHB_2t and b) SDB_2t.

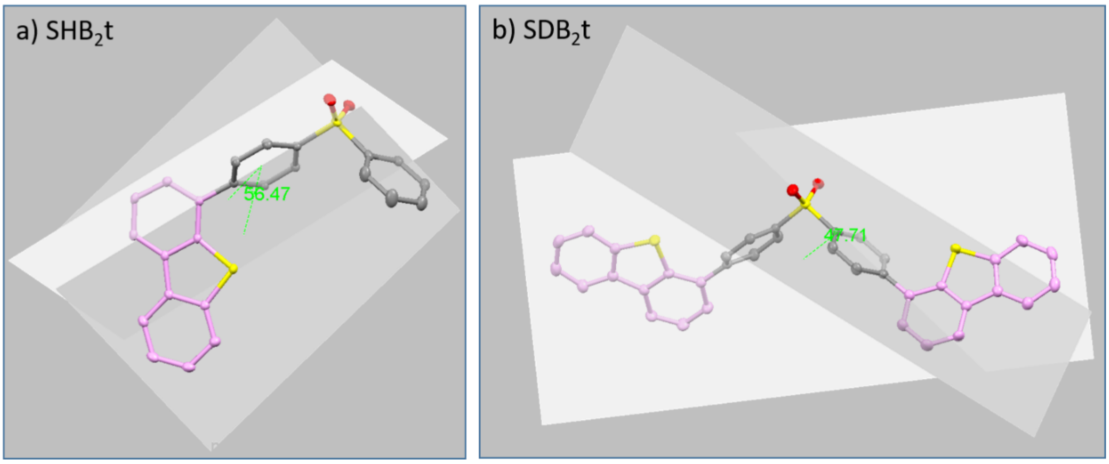

Fig. S13 Dihedral angles between the dibenzothiophene and its adjacent phenyl ring for the molecules of a) SHB_2t and b) SDB_2t in single crystal structures.

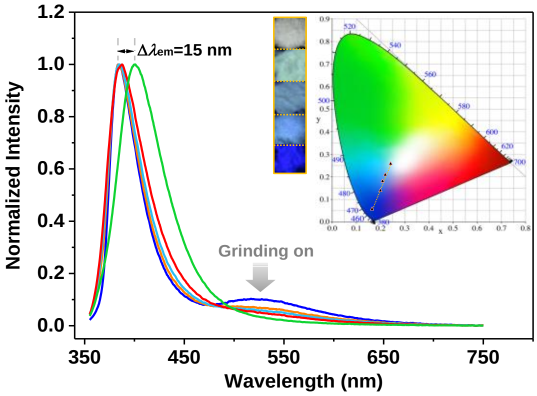

Fig. S14 Changes of PL spectra for SHB_2t upon grinding.

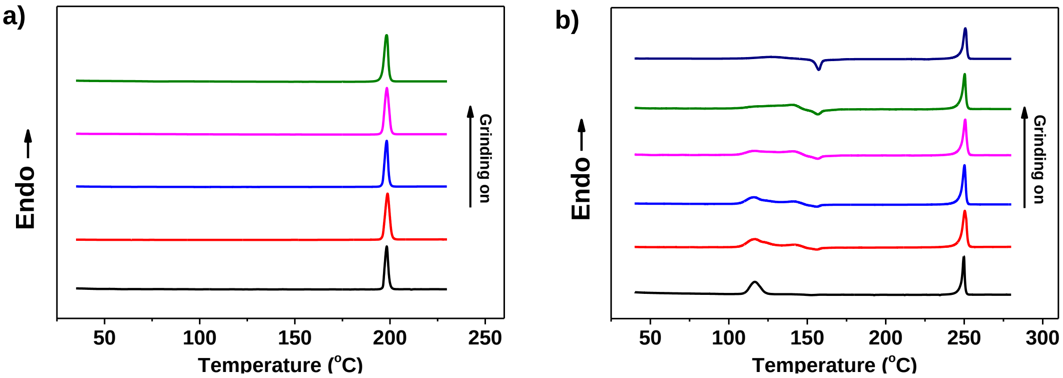

Fig. S15 DSC curves of a) SHB_2t and b) SDB_2t upon grinding.

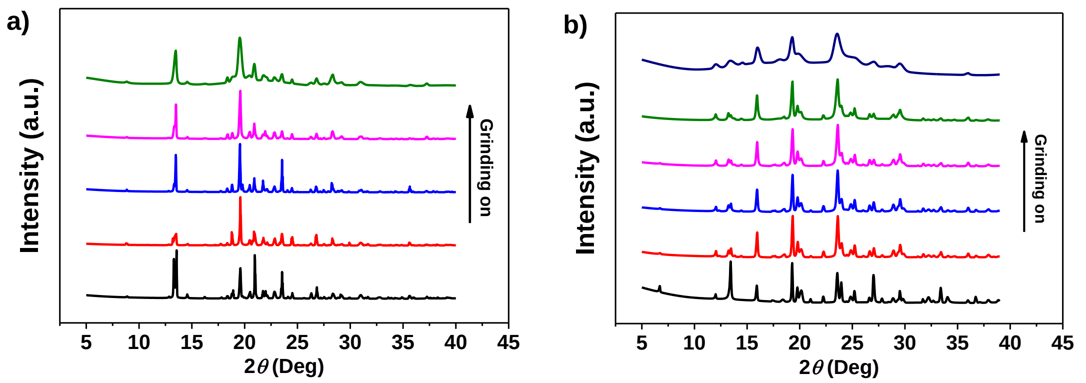

Fig. S16 XRD patterns of a) SHB_2t and b) SDB_2t upon grinding.

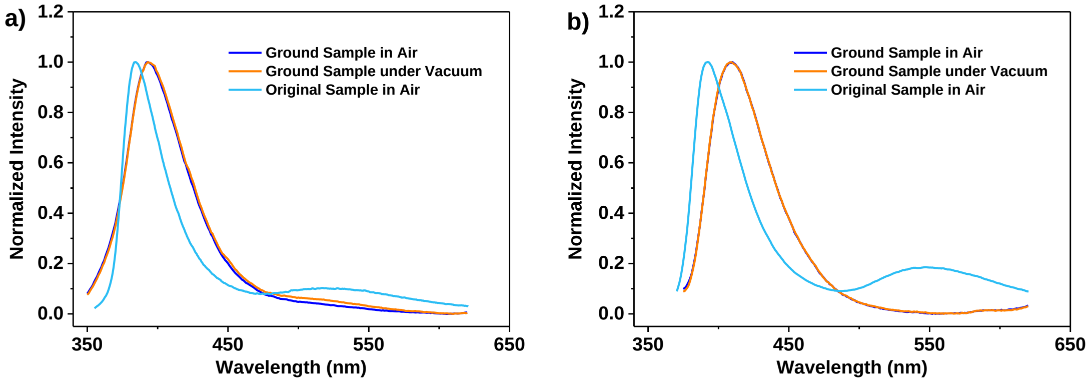

Fig. S17 PL emission spectra of a) SHB_2t and b) SDB_2t under different atmospheric conditions.

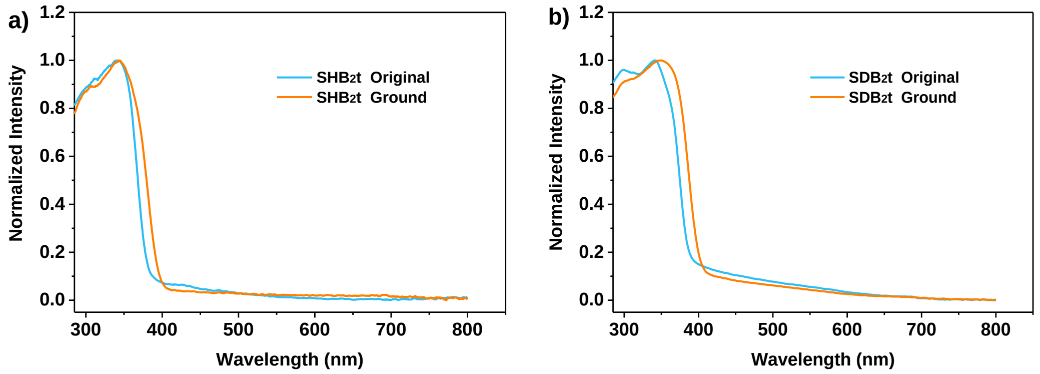

Fig. S18 UV-vis absorption spectra of the original and ground samples for a) SHB_2t and b) SDB_2t.

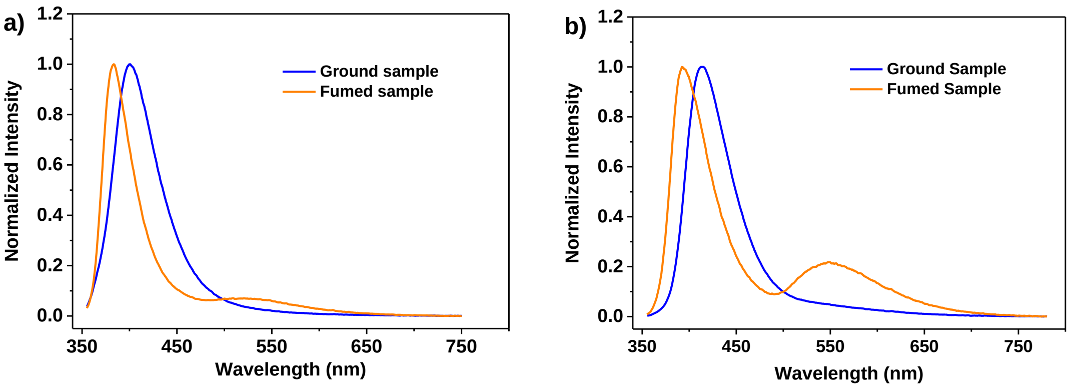

Fig. S19 PL spectra of the ground and fumed samples of a) SHB_2t and b) SDB_2t.

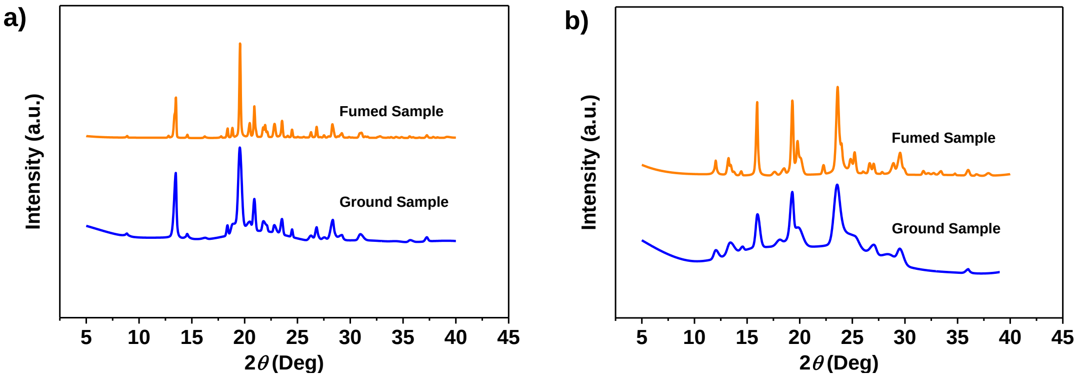

Fig. S20 XRD patterns of the ground and fumed samples of a) SHB_2t and b) SDB_2t.

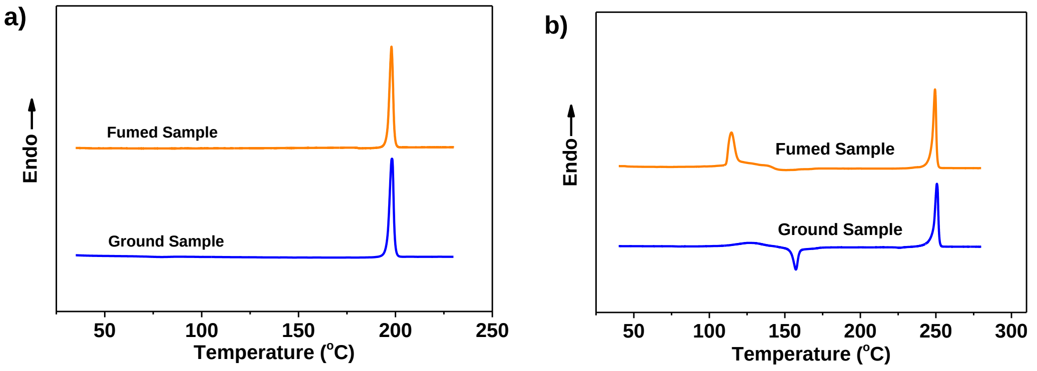

Fig. S21 DSC curves of the ground and fumed samples of a) SHB_2t and b) SDB_2t.

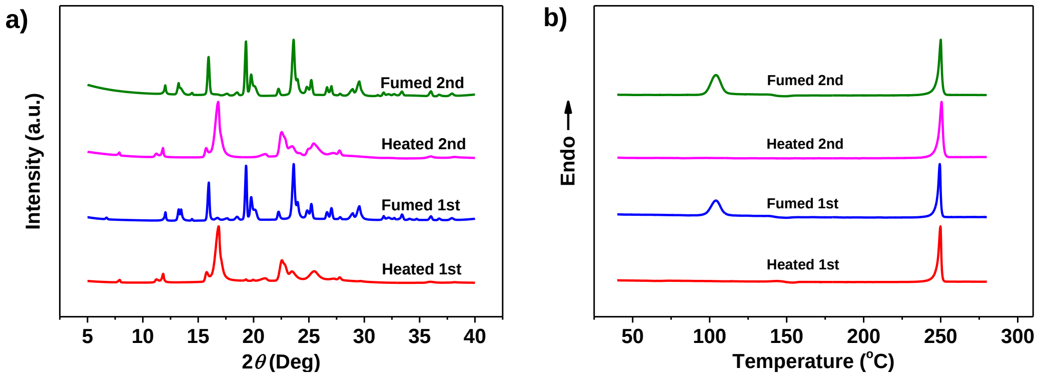

Fig. S22 a) XRD patterns and b) DSC curves of SDB_2t upon heating at 130 °C for about 1 min and fuming in DCM vapor for about 20 min.

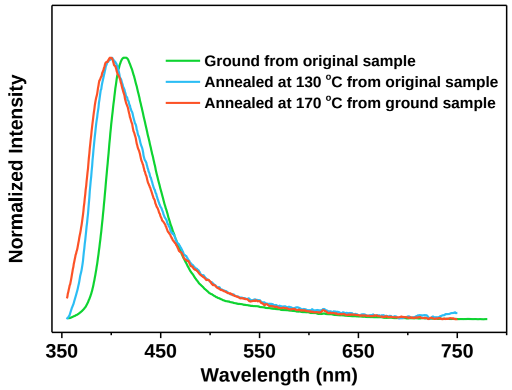

Fig. S23 PL spectra of SDB_2t upon different treatments.

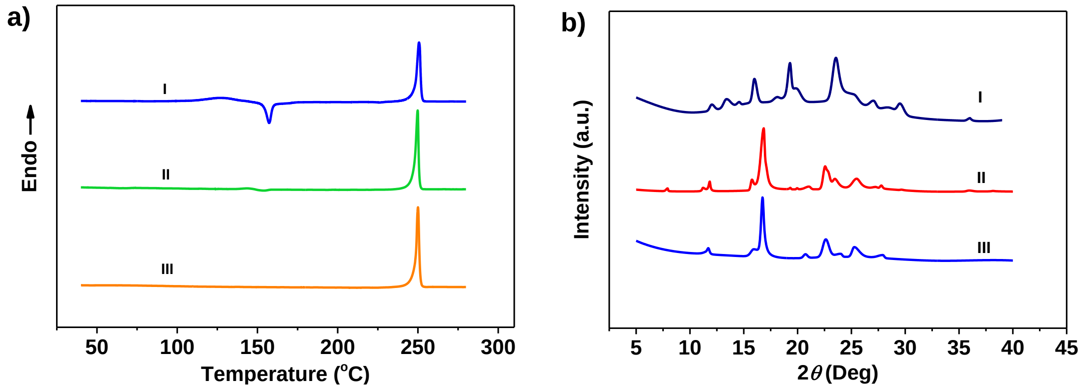

Fig. S24 a) DSC curves and b) XRD patterns of the SDB_2t samples obtained from different treatments. I) ground from original powder; II) annealed at 130 °C for about 1 min from original powder; III) annealed at 170 °C for about 2 min from ground sample.

4. Structural Information

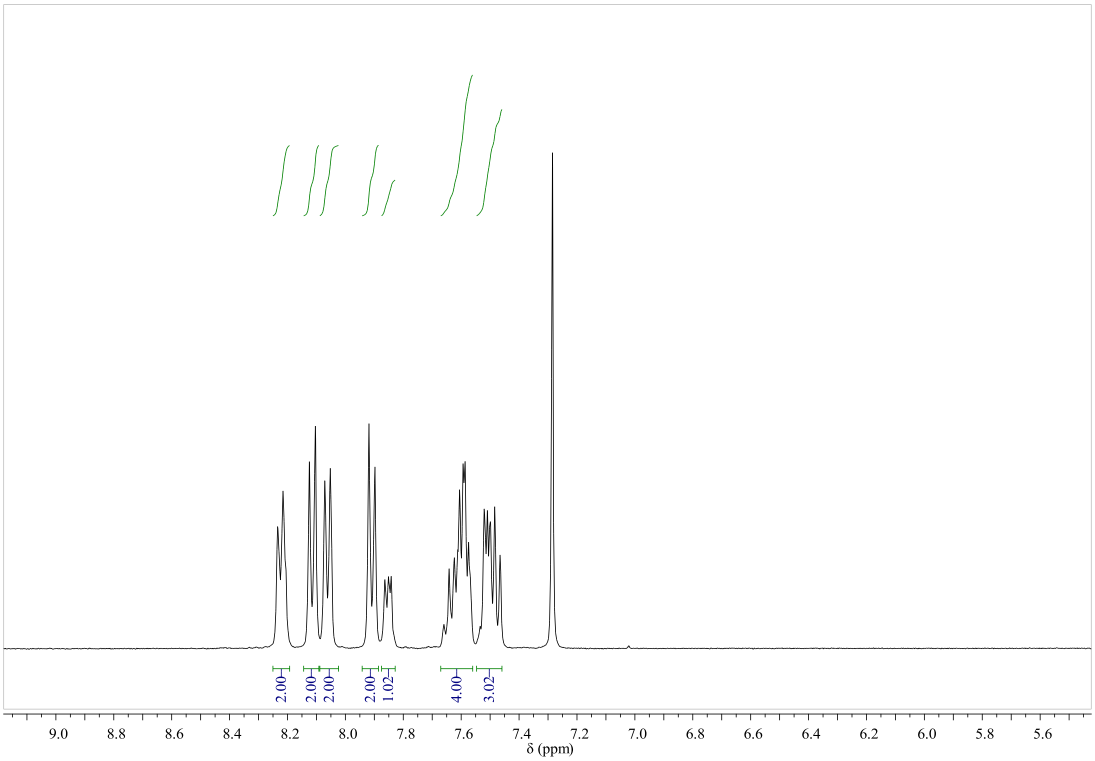

Fig. S25 ^{1}H NMR spectrum of SHB_2t (in $CDCl_3$).

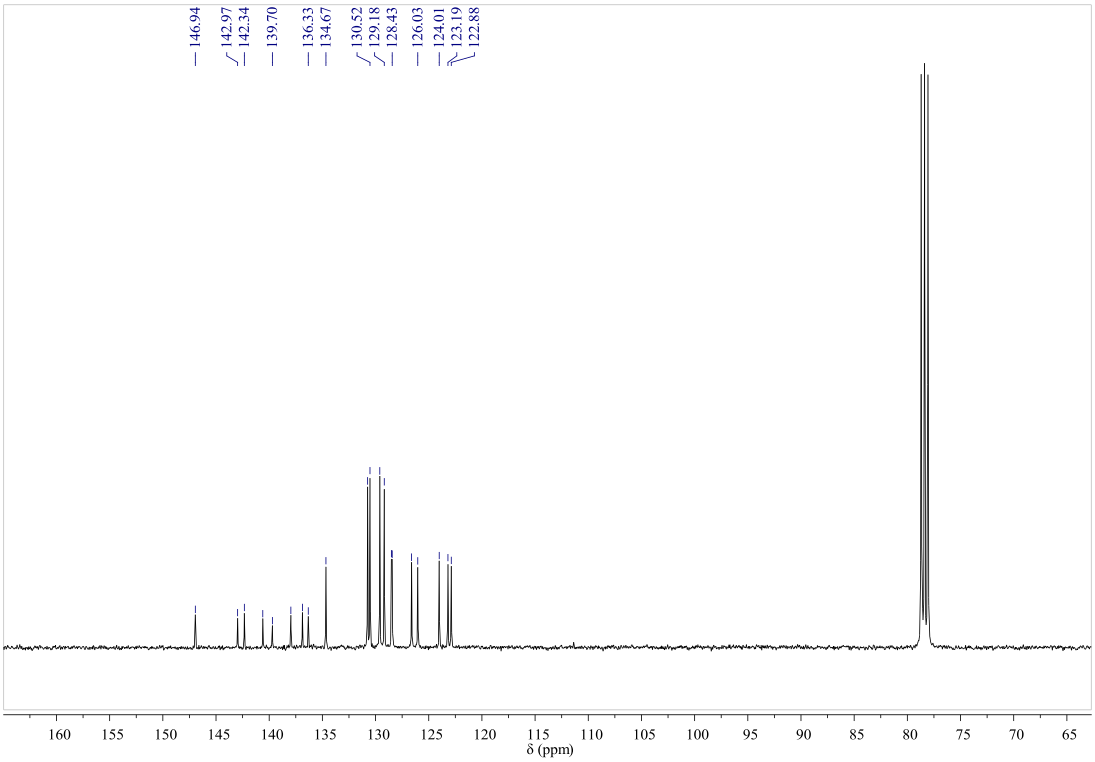

Fig. S26 ^{13}C NMR spectrum of SHB_2t (in $CDCl_3$).

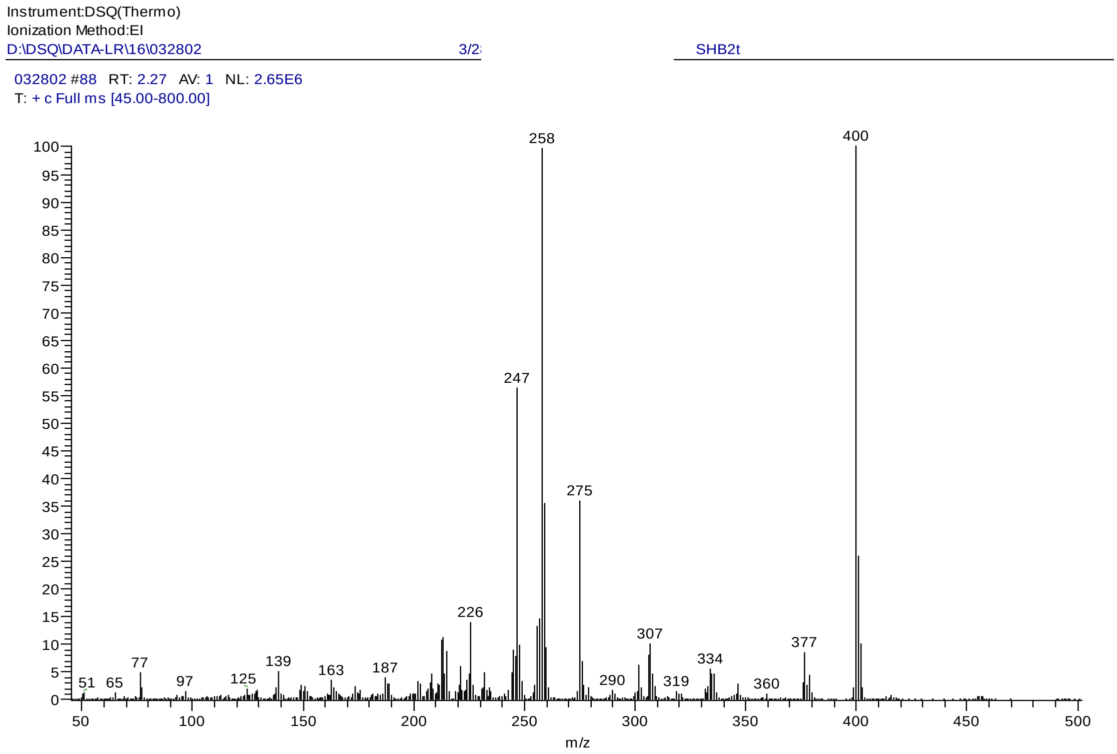

Fig. S27 EI-MS of SHB_2t.

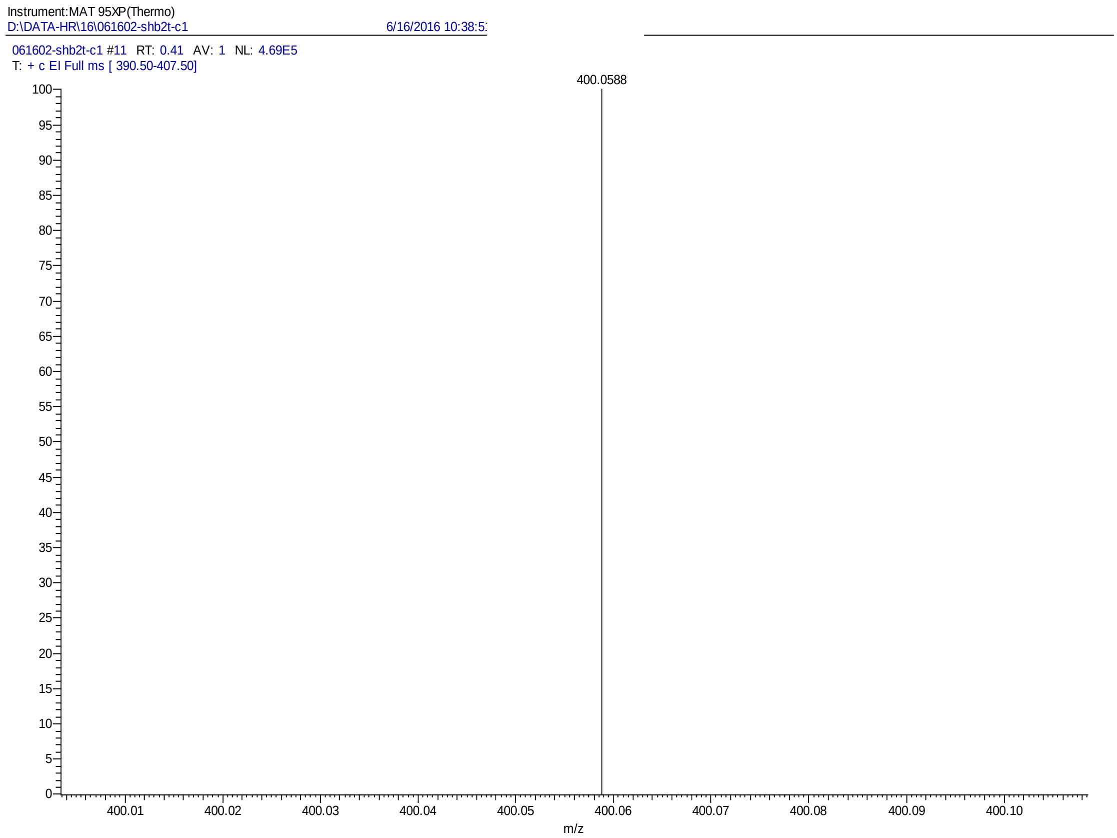

Fig. S28 HRMS of SHB_2t.

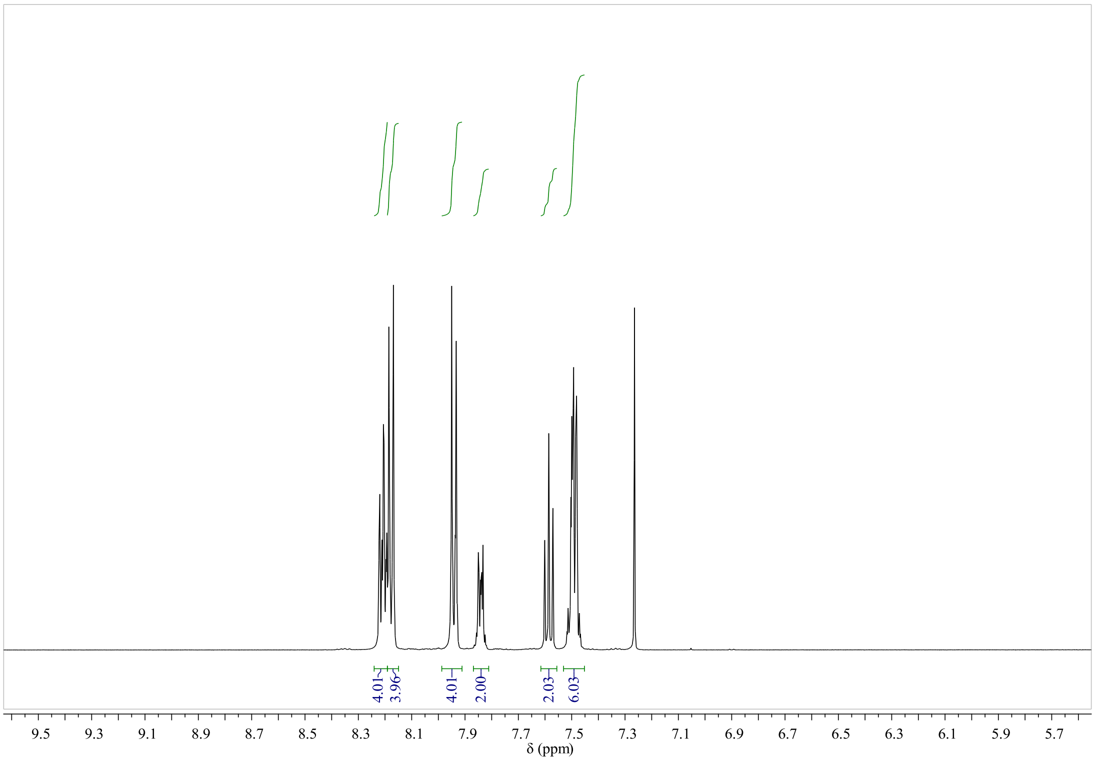

Fig. S29 ^{1}H NMR spectrum of SDB_2t (in $CDCl_3$).

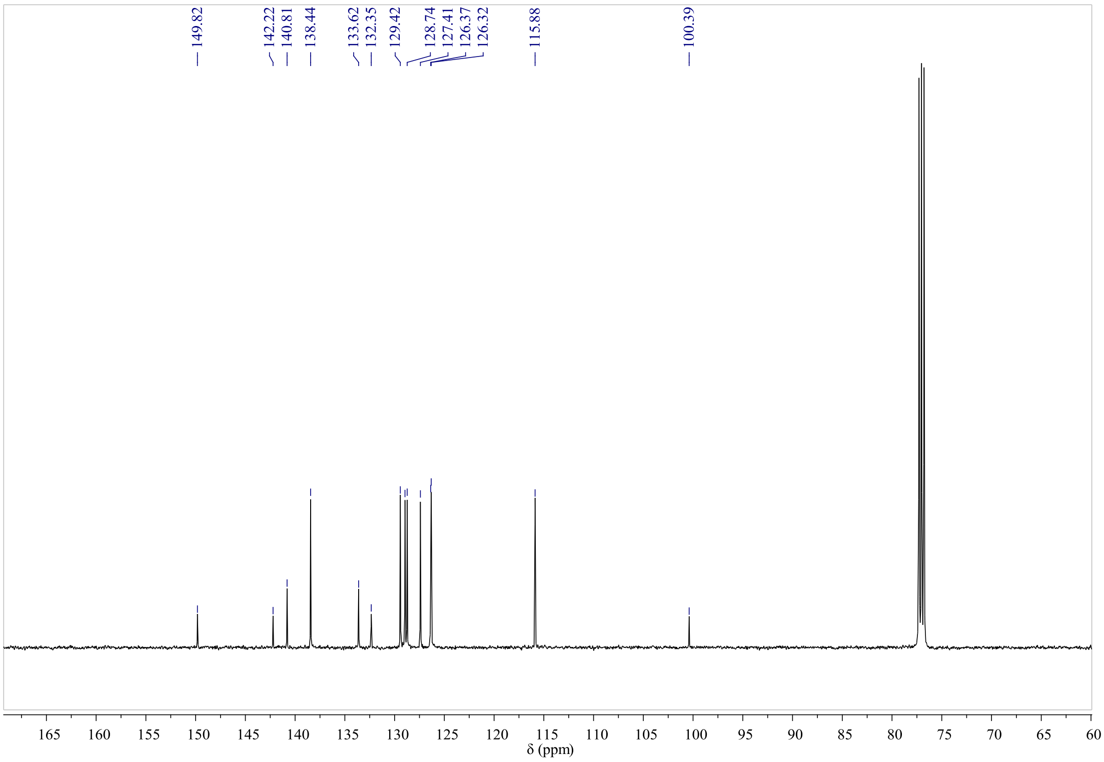

Fig. S30 ^{13}C NMR spectrum of SDB_2t (in $CDCl_3$).

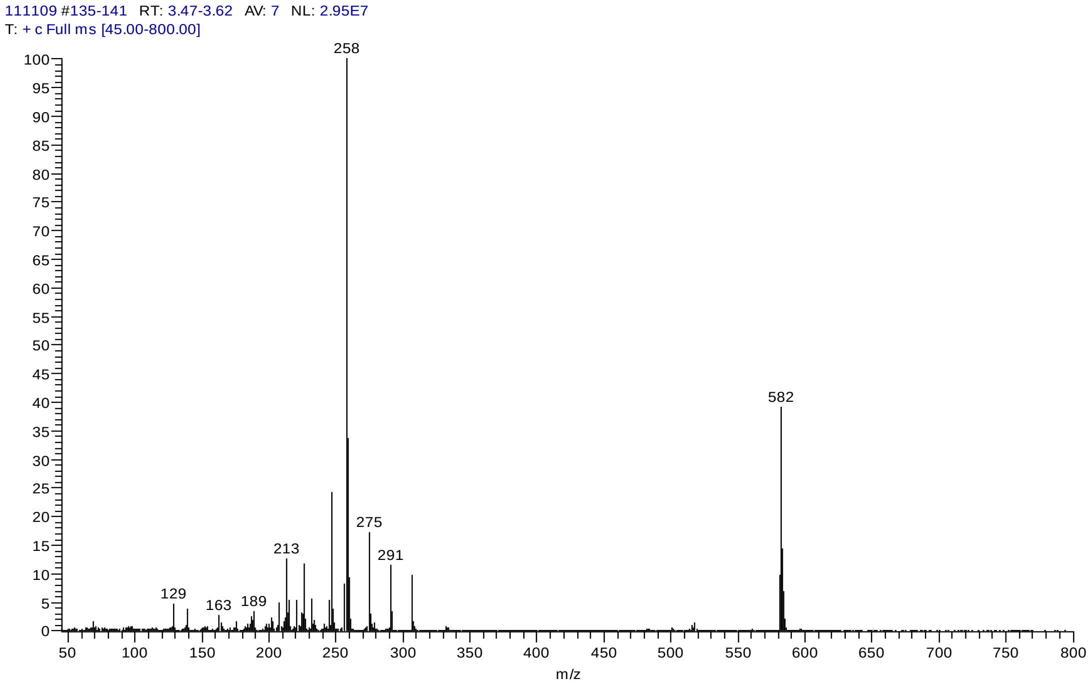

Fig. S31 EI-MS of SDB_2t.

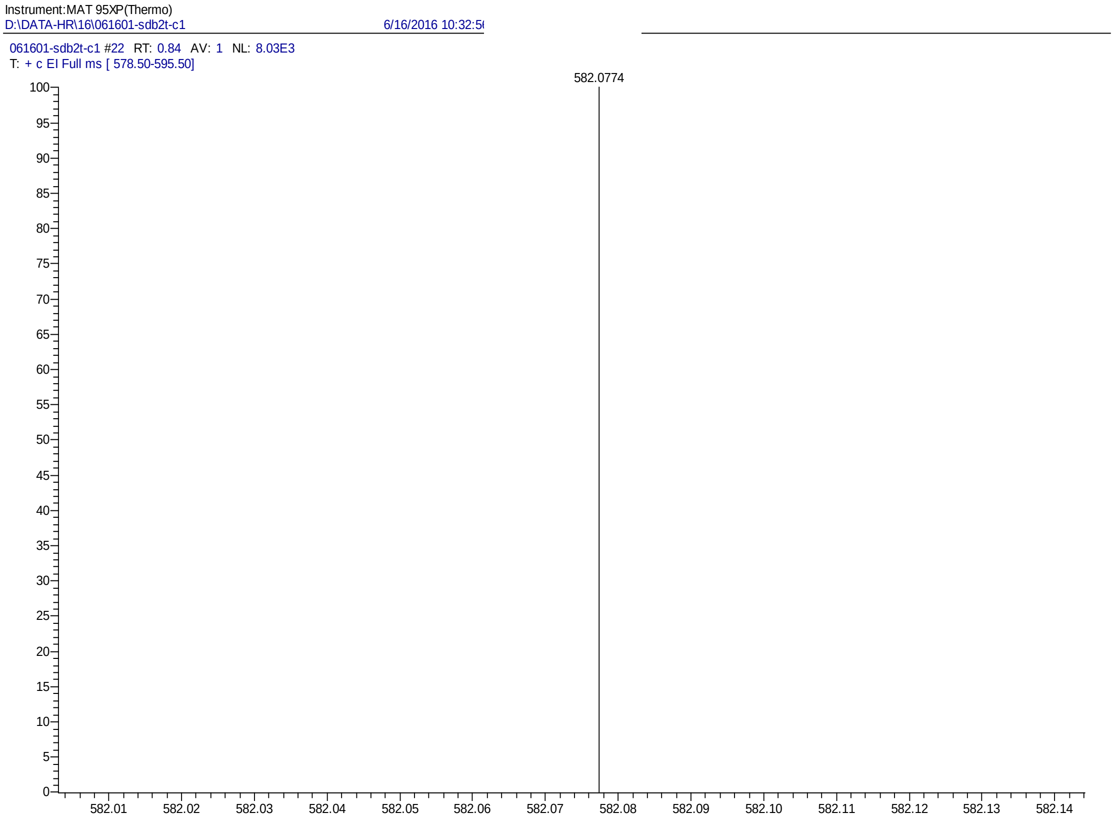

Fig. S32 HRMS of SDB_2t.
